# Current State of PhD Nursing Education in Four Countries: A Comparative Analysis

**DOI:** 10.1177/23779608251395097

**Published:** 2025-12-23

**Authors:** Thóra B. Hafsteinsdóttir, Joseph Almazan, Johanna Heikkilä, Lisbeth Fagerström, Aurelija Blaževičienė

**Affiliations:** 1 Nursing Science Department, Julius Center for Health Sciences and Primary Care, University Medical Center Utrecht, Utrecht, the Netherlands; 2 Medicine Department, School of Medicine, 204569 Nazarbayev University, Astana, Kazakhstan; 3 Institute of Health, School of Health and Social Studies, JAMK University of Applied Sciences, Jyväskylä, Finland; 4 Faculty of Education and Welfare Studies, Health Sciences, 8130 Solutions for Health Academy, Vaasa, Finland; 5 Faculty of Nursing, Academy of Medicine, Lithuanian University of Health Sciences, Kaunas, Lithuania

**Keywords:** doctoral education, PhD education, PhD students, nursing, organizational structure, entry requirements, supervision, mentoring

## Abstract

**Introduction:**

**:** The academic preparation of nurses is crucial to meet the needs of the global population. Doctoral education is imperative for nurses to conduct research and to develop evidence-based practices to ensure that nurses contribute to the health of populations. There are considerable differences between countries in the PhD nursing programs offered.

**Objective:**

To describe the state of PhD nursing education in three West-European countries, including Finland, Lithuania, and the Netherlands, as compared to the Central-Asian Republic of Kazakhstan in terms of organizational structure, entry requirements, supervision, and the required competencies.

**Methods:**

A descriptive survey design was used, including a comparative analysis of data extracted from PhD nursing programs of universities in Finland, Lithuania, the Netherlands, and Kazakhstan.

**Results:**

Although the study showed similarities between the countries, like all countries offer research-oriented PhD programs for nurses, the following differences were identified: The organizational structure is different, as PhD nursing education in European countries is provided by independent nursing science departments chaired by a professor in nursing, while nurses in Kazakhstan can follow PhD education from other disciplines. PhD students in European countries have supervisors with strong expertise in nursing science and research, while supervisors in Kazakhstan are from other disciplines, lacking nursing science expertise.

**Conclusion:**

PhD supervisors’ lack of expertise in nursing science threatens the development of PhD education in nursing in Kazakhstan. Universities in Kazakhstan should develop independent PhD education for nurses with a focus on nursing and to strengthen the supervision of PhD students with well-qualified supervisors having expertise in nursing science. For Kazakhstan to meet the demands of tomorrow's health care, it is critical to develop a greater pool of PhD-prepared nurses with strong competencies in nursing, health care, research, and supervision, to educate a sustainable nursing workforce that contributes to the health and well-being of the Kazakhstani population.

## Introduction

Worldwide, the academic preparation of future nurses is crucial to meet the health care needs of individuals, families, communities, and societies. Nurses are critical for the generation of new knowledge to advance nursing practice, improve healthcare quality, shape health policy, and improve population health (Buchan et al., 2022; WHO, 2020). The PhD degree, the highest academic degree, prepares nurses to develop and translate innovative knowledge; to contribute to the development of nursing science; to define its uniqueness; maintain its professional integrity; and educate the next generation of nurses and nurse scientists (American Academy of Colleges of Nursing (AACN), 2022). In general, two models of PhD programs in nursing are known worldwide. In the research-based European model, PhD students focus on conducting (implementing) research projects which also includes following methodological course work, while the programmatic North American model, PhD students first take courses that are related to the subject of the dissertation and, after successfully passing relevant exams; they concentrate on doing their dissertations (Ketefian, 2001; Ketefian et al., 2005).

## Review of Literature

Research-focused doctoral education in nursing is imperative for nurses to conduct research and to develop advanced evidence-based clinical practices, to ensure that the nursing workforce contributes to the health and well-being of populations, as well as to ensure a vibrant and sustainable nursing workforce (Kim et al., 2022). A research-focused PhD degree prepares nurses to establish, translate, and disseminate new knowledge as leaders within higher education institutions, as well as within the health service system (Broome et al., 2023). PhD-prepared nurses play a critical role in educating and mentoring clinical nurses to provide high-quality and safe care. They often work as faculty members at universities and thereby have an important role in teaching (under)graduate nurses and nurse scientists (American Academy of Colleges of Nursing (AACN), 2022; Kim et al., 2022; National Academy of Sciences, Engineering, and Medicine (NASEM), 2021). Internationally, a PhD degree is the main criterion required for academic staff leading master's and PhD programs (AACN, 2022). Although the number of PhD-prepared nurses worldwide is not known due to a lack of studies in this area (Dobrowolska et al., 2021), it is estimated that in most countries < 2% of nurses hold a PhD degree (Cheraghi et al., 2014; NASEM, 2021). Despite the growing number of PhD programs in nursing (Dobrowolska et al., 2021), there is still a limited number of PhD-prepared nurses to respond to the extensive challenges in health care (NASEM, 2021). Therefore, there is a pressing need to expand the PhD-prepared nursing workforce globally.

Doctoral degrees in nursing have been offered in the United States since 1933, with increasing interest emerging in the 1970s with the establishment of nursing faculties at universities (Carter, 2013; Hafsteinsdóttir et al., 2019; Ketefian & Redman, 2015; Reid Ponte et al., 2015). In Europe, doctoral nursing education has developed mainly in the last decades. The 1999 Bologna Process supported the European Higher Education Area (EHEA), promoting standardized higher education across EU countries (European Union, 2022) and fostering doctoral education growth (Lahtinen et al., 2014). Countries like Finland, Lithuania, and the Netherlands have well-established nursing education. In Finland, universities of applied sciences (UAS) offer 3.5-year bachelor's programs and two types of master's programs, which can either be a research orientated Master of Science at universities or research and practice based Master within UAS, while universities provide PhD degrees (Leino-Kilpi & Stolt, 2019). PhD education, introduced in 1979, is tuition-free for EU citizens. PhD students are supported through a national collaborative network in nursing science (Finnish Doctoral Education Network in Nursing Science) in close collaboration with the Baltic/Nordic network, and the European Academy of Nursing Science (EANS, 2024), to provide doctoral summer schools for PhD students. A national postdoctoral nursing program further supports PhD-prepared nurses (Kim et al., 2022). In Lithuania, colleges offer three-year undergraduate programs, while universities provide four-year bachelor's degrees, along with master's and PhD programs. In the Netherlands, universities of applied sciences offer four-year bachelor's degrees and two-year Master of Advanced Nursing Practice programs. A two-year Master of Nursing Science program is offered at one university, and most universities offer PhD training for nurses (Lahtinen et al., 2014), with many PhD students attending the EANS doctoral summer school (EANS, 2024). A national leadership and mentoring postdoctoral nursing program is offered to PhD-prepared nurses (Hafsteinsdóttir et al., 2020).

In Central Asia, only Kazakhstan and Uzbekistan offer master's and PhD programs in nursing (Llop-Gironés et al., 2024). Despite Kazakhstan signing the Bologna Declaration in 2010 (European Commission, 2024), master's and PhD education in nursing remain in the early stages (Järvinen et al., 2023). The master's program began in 2015, and PhD education for nurses was introduced in 2020 (Heikkilä et al., 2021; Järvinen et al., 2023). Kazakhstan offers a three-year college program leading to “general practice nurse” (Lahtinen et al., 2014), a 3.5-year applied bachelor's program at European Qualification Level 5, and a four-year bachelor's program at medical universities (Order of the Minister of Health of Kazakhstan, 2022). Until recently, PhD opportunities for nurses were not available to nurses.

In European countries, PhD nursing education is research-focused and often includes coursework leading to the final research thesis (Dobrowolska et al., 2021), with the content of programs, organization, entry requirements, and competencies to be developed differing between countries (Dobrowolska et al., 2021; Lahtinen et al., 2014; McKenna et al., 2014). In some countries, PhD students are supervised by supervisory teams including highly qualified academic professionals, like professors in nursing, while in other countries, the number of supervisors may be limited, and they lack academic qualifications. Moreover, in the different roles that PhD-prepared nurses work, they are expected to have a wide range of competencies in research, clinical practice, and teaching for different settings. The development of competencies during the academic development requires ongoing assessment, starting with the PhD trajectory (Smaldone & Larson, 2021), which is important for each doctoral candidate.

Ensuring continuous quality assurance and monitoring is crucial for strengthening PhD nursing education. High-quality doctoral programs and regular evaluations are essential for advancing the profession (Breslin et al., 2015; Smeltzer et al., 2015). European universities have improved PhD programs by fostering a culture of quality involving university leadership, faculty, staff, and students, emphasizing accountability and enhancement (Hasgall et al., 2019). Key factors in high-quality PhD nursing education include supportive academic environments and PhD-prepared faculty who are active researchers and mentors (Minnick et al., 2017; Rollins Gantz & Hafsteinsdóttir, 2023; Smeltzer et al., 2015; Volkert et al., 2018).

Järvinen et al. (2023) investigated the status and improvements made by universities in Kazakhstan in nursing research infrastructure in terms of: library, internationalization, finance, information and communication technology, and research, development, and innovation. The findings showed that Kazakhstani universities providing nursing education are still in the process of developing their nursing research infrastructure and have not acquired access to nursing databases, with libraries lacking textbooks on nursing research. None of the universities had joined international nursing networks. Although participation of university staff and students in nursing conferences had increased, no investment was made in nursing research projects (Järvinen et al., 2023). Therefore, despite the global growth of doctoral nursing programs, many countries still lack such programs or face challenges in the development or implementation of programs, resulting in a limited number of PhD-prepared nurses.

This study aimed to compare and analyze the PhD nursing education in Finland, Lithuania, the Netherlands, and Kazakhstan to address similarities and differences in the PhD nursing education offered in terms of the organization of programs, entry requirements, supervision, as well as competencies students need to develop.

## Method

### Design

This study used a descriptive design including a survey and a descriptive comparative analysis of data extracted from selected PhD nursing programs from Nursing Science Departments (NSD) in four countries. The study was part of an Erasmus + funded capacity building project carried out in four countries: Finland (FI), the Netherlands (NL), Lithuania (LI), and Kazakhstan (KZ). Three of the four countries (FI, NL, and LI) have long-established PhD nursing educational programs and a strong nursing science background, while Kazakhstan recently launched PhD nursing education with limited nursing science capacity. The original data was collected between February and April 2021.

### Research Questions

The research questions were the following:

1. What are the organizational structures of PhD nursing studies in the participating countries?
2. How is the supervision system for PhD nursing studies established in the participating countries?
3. What are the entry requirements for a PhD and the requirements leading to the final PhD degree in the participating countries?
4. What are the core competencies that PhD students need to develop, and how are they evaluated in the participating countries?

### The Sample

The study was conducted in countries that participated in an Erasmus + collaboration project, intended to strengthen capacity building in nursing science. Therefore, the sample is purposive, including NSD overseeing PhD nursing education of a university from each of the four countries: FI, NL, LI, and two universities in KZ.

### Data Collection and Analysis

Data were collected by the international research team of researchers and academic scholars, who were contact persons in the participating departments, on the organizational structure, the supervision system, requirements for the PhD degree, and the core competencies that PhD students need to develop, and how these are evaluated.

The survey questionnaire was self-developed by the research group concerning the organizational issues at the program level, as no such questionnaire was identified in the literature. The face validity of the survey questionnaire was determined by the research team, who are experienced researchers and academic scholars. The research team reviewed the questionnaire on the following aspects: relevance: if the questions related to the variable being measured; clarity: if the questions were easy to understand and free of jargon; appropriateness: if the format and language of the questionnaire was suitable for the intended audience; and formatting: if the items were presented in a clear and logical way. The research team met regularly to ensure common understanding and consensus on the content of the questionnaire (Polit & Beck, 2017). Further psychometric testing was not conducted. The survey included 29 questions about (a) the organizational structure of PhD nursing studies; (b) the supervision system for PhD nursing studies; (c) the admission requirements for PhD studies and requirements leading to the final PhD degree, and (d) the core competencies developed during the PhD training and how they were evaluated in the participating countries. After completion, the gathered information was transferred into an Excel document.

Data analysis included descriptive analysis of numerical data, whereas content analysis was conducted of the narrative data (Polit & Beck, 2017). Thereby, similarities and differences between the PhD programs were summarized by two researchers. In the case of inconsistencies, these were discussed, and a consensus agreed on. All team members then read the comparative material, after which necessary clarifications on the details of the programs were provided by the university contact persons and supplemented to the summary and analysis until an agreement was reached. To maintain data consistency across different linguistic contexts, a series of standardized procedures were used. Textual data were first translated, using a forward–backward translation, conducted by bilingual and multilingual experts to ensure semantic and conceptual consistency. A uniform coding framework and a codebook were developed by the researchers to guide data categorization and interpretation across languages. To enhance reliability, independent reviewers performed cross-linguistic validation by comparing randomly selected data samples and assessing intercoder agreement. These procedures collectively ensured that data collected in multiple languages remained comparable, accurate, and analytically consistent throughout the study.

### Ethical Considerations

All participating institution members were part of the Erasmus + funded capacity building project collaboration, and all project partners had signed a partnership agreement approving the activities, including this research. The study was part of this collaboration and was based on public information. Therefore, approval to conduct the study was not needed from the participating institutions.

## Results

### The PhD Programs and Organizational Structure

The PhD programs in the participating countries were all reported to be research-oriented and offered at universities. The number of universities offering PhD nursing education ranged from five universities in Finland and the Netherlands to one university in Lithuania and two universities in Kazakhstan at the time of the study. In Finland, Lithuania, and the Netherlands, PhD nursing education was reported to be specific for nurses and led by the head of the NSD, a university professor in nursing science. In Kazakhstan, nurses could follow the PhD training offered by other disciplines, led by a university professor of that specific discipline (e.g., public health) without expertise in nursing science.

The PhD programs had different types of positions and funding: in Finland, PhD students in nursing had a full-time or part-time position, which could be with or without a scholarship. The length was generally 4 years, but it could be up to eight years. Lithuania offered PhD programs in nursing with or without a scholarship for a length of 4 years. In the Netherlands, PhD training was generally 4 years, and PhD students either had an employee status, which could include a scholarship or external funding, or the student could be an external PhD candidate (Brinke ten & Walsum van, 2020). Although Kazakhstan was reported to offer a three-year PhD program with state grants, the majority of universities offered four-year programs (Order of the Minister of Education and Science of the Republic of Kazakhstan, 2018).

### Entry Requirements for PhD Programs

In all the participating countries, the master’s degree was reported to be an entry requirement to PhD programs, referring to the International Standard Classification of Education (ISCED, 2011). In the Netherlands, PhD candidates further have to meet stringent academic requirements, possess a solid background in the theory and methods within the field of nursing science, and demonstrate a good knowledge of English.

Although the content of PhD programs varied between and within countries, in all participating countries, PhD students were required to conduct original research in the field of nursing and to follow theoretical and methodological courses. Besides conducting research, in the Netherlands, PhD students followed courses in thematic research areas, professional development, and had teaching responsibilities. In Kazakhstan*,* the PhD programs included theoretical coursework and teaching responsibilities (Table 1).

**Table 1.** Organizational Structure of PhD Studies Within the University.

Country	Institute type awarding a PhD degree	The number of universities that can award a PhD degree in nursing	PhD program chair	Entry requirements for the PhD program	Types of positions for a PhD candidate and funding	ECTS^b^	Time to complete PhD studies	Content of PhD programs
Finland	University	Five universities	Head of the department, professor in Caring Science	Master's degree, ISCED^a^ level 7	PhD candidate (a) with a full-time or part-time position; (b) with scholarship; (c) without a scholarship.	240 ECTS	4 years are recommended time (b) and (c) are 6–8 years	Content PhD programs varies depending on research focus; they generally include theoretical and methodological courses.
Kazakhstan	University	Two universities in 2021; five in 2025	University professor with an independent research program.	Master's degree, ISCED^a^ level 7, and at least 9 months of practical work experience.	PhD candidate with a state grant with a half-time position.	240 ECTS	3–4 years	The PhD programs include theoretical coursework, teaching responsibilities, and the dissertation defense completion.
Lithuania	University	One university	University professor from the nursing field	Master's degree; ISCED^a^ level 7	PhD candidate (a) with scholarship; (b) without a scholarship.	NA	4 years	PhD programs focus on conducting original research in the nursing field. Generally includes theoretical and methodological courses depending on the area of research.
The Netherlands	University	Five universities	University professor with an independent research program in nursing science and head of the nursing science department.	Solid knowledge in theory and research methods; master's degree; ISCED^a^ level 7; demonstrate good knowledge of English	PhD candidate (a) with employee status; (b) with scholarship; (c) externally funded; (d) external PhD candidate. ISCED level 8.	NA	4 years	PhD programs focus on conducting original research. Generally, include theoretical and methodological courses depending on the research area and professional development.

^a^ ISCED refers to the International Standard Classification of Education 2011 (ISCED, 2011).

^b^ ECTS refers to the European Credit Transfer System (European Credit Transfer and Accumulation System). One ECTS credit is 27–30 hr of students' work.

### Supervision of PhD Students

The supervision of PhD nursing students was conducted by a supervisory committee. In Finland, the supervisory committee was chaired by a university professor in nursing science. In the Netherlands, the supervisory committee included two to four members and was led by a main supervisor, a university professor in nursing science. In Lithuania, the supervisory committee included eight university professors from nursing science and two from public health. In Kazakhstan, PhD students were supervised by at least two supervisors appointed by the university's order. The main supervisor was from the same institution where the PhD candidate was enrolled, while the co-supervisor was from another country.

For PhD supervision in nursing in Finland, the minimum required qualification level was associate professor competence. In Lithuania, the chair of the committee was a professor with the qualification Doctor of Nursing Science. In the Netherlands, the qualification requirement for PhD supervision was a professor or associate professor (Hammerslag et al., 2025). In Kazakhstan, the academic supervisors were required to have a scientific degree corresponding to the profile of the requested direction (Order of the Minister of Education and Science of the Republic of Kazakhstan, 2018).

The supervisory committee had the role of supervising students during their PhD journeys. In Finland, the supervisory committee was required to provide high-quality supervision and to guarantee the progress of the PhD student's research and the development of research competencies. In Lithuania, the supervisory committee regularly assessed the research conducted by a PhD student. In the Netherlands, the supervisory committee was required to provide high-quality supervision to the PhD student in conducting the research and developing his/her research competencies, and further to evaluate whether manuscripts met the requirements. In Kazakhstan, the supervisory committee was required to guide the student throughout the research journey and to provide recommendations for improvements, ultimately ensuring that the research meets academic standards and contributes to the field (Table 2).

**Table 2.** Supervision of PhD Students.

Country	Supervisory committee (yes/no)	Members of the supervisory committee	The qualifications of supervisors	The role of the PhD-supervisory committee:
Finland	Yes	The main supervisor is a university professor and has a position at the home institute. The co-supervisor is at least an associate professor and can be from the same or another department, or external.	The minimum required level is associate professor competence.	To provide high-quality supervision. To guarantee the progress of the doctoral student's research and the development of the research competencies.
Kazakhstan	Yes	The principal supervisor is a researcher from a home institution located in the country, while the co-supervisor is from abroad, from partner universities. Chairman of the State Certification Committee and members of the State Certification Committee (SCC).	Academic supervisors must meet the following requirements: - Have a scientific degree corresponding to the profile of the requested direction. - Have experience in scientific and pedagogical work for at least three years. - Author of at least five scientific articles over the past 5 years in publications included in the list of scientific publications recommended for the publication of the main results of scientific activity, approved by the authorized body in the field of education and science, and one scientific article in an international peer-reviewed scientific journal that has an impact factor according to JCR data.	Generally, the PhD supervisory committee guides and supports the student throughout the research journey. The committee helps define the research direction, provides constructive feedback, and ensures the project is on track. Members offer expertise in relevant fields, advising on methodology, literature, and theoretical frameworks. They also monitor the student's progress, addressing any challenges or issues that may arise. Additionally, the committee evaluates the student's work at various stages, providing recommendations for improvements. Ultimately, the supervisory committee plays a crucial role in ensuring the research meets academic standards and contributes to the field.
Lithuania	Yes	Eight university professors from the nursing field and two professors from the public health field.	The chair of the committee is a Professor nominated by the Dean and approved by the Research Council, and has the following qualifications: - is a doctor of nursing science; - an appointment at LSMU; - senior researcher; - in-depth knowledge of the discipline; - demonstrable high-level recognition of academic publications; - The development of research programs; - the ability to train researchers; - the management of research alliances; - in-depth insight into research quality assurance; - knowledge of the interrelationship between disciplines.	The supervisory committee conducts regular assessments of the research conducted by PhD students in the faculty and provides supervision to the PhD candidate during the PhD journey.
The Netherlands	Yes	University professor and at least two other (associate) professors, all with a PhD degree and experience in supervising.	Professor or associate professor, nominated by the Dean following consultations with the chair holder, and has the minimum qualifications: - is a doctor of philosophy degree; - an appointment at Utrecht University or at UMCU; - senior researcher; - in-depth knowledge of the discipline; - demonstrable high-level recognition of academic publications; - the development of research programs; - the ability to train researchers; - the management of research alliances; - in-depth insight into research quality assurance; - knowledge of the interrelationship between disciplines.	The supervisory committee has the role of supervising the PhD student during the PhD journey and conducts regular assessments of the research conducted and manuscripts reporting on studies included in the PhD thesis. A Training and Supervision Agreement is set up outlining the objectives of the research, including an individualized plan of training and supervision of the PhD student.

### Requirements Leading to the PhD Degree

In Finland, earning a PhD in nursing requires completing a written thesis summary along with three to four research articles, at least one of which must be published or accepted for publication. Alternatively, the thesis could be presented in the form of a monograph. Additionally, PhD candidates need to complete a theoretical coursework program. In the Netherlands, the final PhD thesis was required to demonstrate the candidate's scientific development, reporting on a range of studies conducted by the student in collaboration with his supervisory team, which includes a general introduction, a number of research chapters based on a range of research articles, forming a coherent line of research. Although the number of scientific papers required for the thesis varied, generally three or more scientific papers were required. PhD students were also required to follow methodological courses in line with their research studies. In Lithuania, the requirement for the PhD degree in nursing was composing a monograph, publishing at least two articles with a citation index, and presenting the findings at two international conferences. In Kazakhstan, the PhD thesis was required to include a manuscript and two to three scientific papers (Table 3).

**Table 3.** Requirements Leading to the PhD Degree.

Country	The aim	The PhD thesis: (a) a range of scientific papers reporting on a coherent line of studies which form a book; (b) a monograph	Each chapter reports	At the end of PhD studies: thesis and/or papers published Other requirements: - presentations; - courses followed; - other
Finland		The PhD thesis includes a recommended three to four scientific papers and a summary. A PhD thesis as a monography is accepted in specific cases.	Each research chapter reports on research conducted, demonstrating and reporting in line with the IMRAD^a^ structure and includes the following sections: (a) identifying a gap in scientific knowledge; (b) outlining design/method; (c) describing data collection and analysis of data, results, and reflecting on the results within the context of the specific field; (d) limitation section.	At the end of the PhD studies, the student must have: (a) At least two articles accepted or published manuscripts in journals that are approved by the national system of publication, forum level 1–3 quality, or with an impact factor. (b) Two manuscripts are in the submission stage.
Kazakhstan	The aim is to train leaders, research scientists, experts, and educators in the field of nursing science, for practical work in educational and scientific organizations in the field of health care.	The PhD thesis includes two to three scientific papers and a manuscript. A PhD thesis as a monography is accepted in specific cases.	At the end of the PhD studies, the student must have: At least one article in the journal of Scopus or Web of Science databases, and at least one article in the journal which recommended by the Science and Higher Education Assurance Committee of the Ministry of Science and Higher Education of the Republic of Kazakhstan	At the end of the PhD studies, the student must have: (1) a manuscript; (2) optional: at least two articles and one review or three articles published in journals included in the and/or first (second) quartile by impact factor according to the Journal Citation Reports of Clarivate Analytics. In one of the articles, the doctoral student is the first author or corresponding author.
Lithuania	The aim is to train scientists of the highest competence in the fields of health sciences, to carry out scientific research aimed at improving the health of people and developing science-based methods of care treatment, and applied to preserve and enhance human health and welfare.	The PhD thesis includes a range of studies reported in scientific papers collated in a book format (b). The PhD thesis includes sections: (a) general introduction; (b) a number of research chapters, including a range of studies, which form a coherent line of research with some published in scientific peer-reviewed journals and some are in the process of publication; and (c) a general discussion.	Each research chapter reports on research conducted demonstrating that the scientific research cycle was followed and reported in line with the IMRAD^a^ structure and includes the following sections: (a) identifying a gap in scientific knowledge; (b) outlining design/method; (c) describing data collection and analysis of data, results, and reflecting on the results within the context of the specific field; and (d) limitation section.	At the end of the PhD studies, the student must have: (a) Two published manuscripts in a journal with an impact factor. (b) Attendance in two international conferences and presented own research data. Optional: Long-term international internship.
The Netherlands	The aim is to train the PhD candidate towards an independent academic and scientist, who is fit for a career inside or outside academia.	The PhD thesis includes scientific papers reporting on a coherent line of studies, which are collated in a book format. The number of papers required is at least three published, with other manuscripts in the publication process. The PhD thesis includes sections: (a) general introduction; (b) a number of chapters includes a range of studies, which form a coherent line of research with some published in scientific peer-reviewed journals and some in the process of publication; and (c) a general discussion.	Each chapter reports on research conducted demonstrating that the scientific research cycle was followed and reported in line with the IMRAD^a^ structure and includes the sections: (a) identifying a gap in scientific knowledge; (b) outlining design/method; and (c) describing data collection and analysis of data, results, and reflecting on the results within the context of the specific field.	At the end of PhD studies, the student must have produced: the PhD thesis, which includes sections: (a) general introduction; (b) a number of chapters, including range of studies, which form a coherent line of research with some published in scientific peer-reviewed journals and some are in the process of publication; and (c) a general discussion. PhD students follow methodological courses supporting the research. Student present studies on research conferences.

^a^ IMRAD structure includes I: Introduction, M: Methods, R: Results, and D: Discussion (Sollaci & Pereira, 2004).

### PhD Competencies and How They Are Assessed

The countries described considerable differences in the competencies required for the PhD degree in nursing and in how they were assessed. Only the Netherlands used a competency model and structural assessment of the competencies required. In Finland, the central competencies required were academic competencies in terms of research skills and methods, deep knowledge in the research topic, and competence in reporting research findings. The assessment of the doctoral thesis focused on conceptual clarity and theoretical mastery of the subject, and on the justification and appropriateness of methodologies and appropriateness in how the methodologies had been used. In Lithuania, the essential competencies for PhD nursing students encompassed a diverse array of skills crucial for success in nursing research and academia. The PhD students’ competencies were assessed through their research, educational endeavors, and clinical practice activities. In the Netherlands, PhD students in nursing developed a range of academic competencies in terms of research methodologies, transferable and personal competencies important for the future academic career, evaluated using the PhD Competence Model to monitor the student's development (Stouthard & Cohen, 2016). In Kazakhstan, diverse assessment methods were used to evaluate key competences for PhD candidates, including research skills as well as skills in multidisciplinary collaboration. The PhD dissertation was assessed, defended orally, complete examinations taken, research findings published and presented (Table 4).

**Table 4.** PhD Core Competencies and How Are They Assessed.

Country	Competencies required (yes/no)	Competence model used	Competence areas required for a PhD	Competence assessment conducted (yes/no)	Competence assessment tool	Competence assessment conducted
Finland	Yes	No	Research skills, knowledge in the research area, and reporting skills	Yes	No	Pre-examination of two external experts. Oral defense. Final assessment of an assessment committee and the opponent.
Kazakhstan	Yes	Thesis evaluation, oral defense, publication, and presentation: Coursework and examinations professional development activities.	Research skills, interdisciplinary collaboration, and professional development.	Yes	No	Thesis evaluation, oral defense: publication and presentation.
Lithuania	Yes	No	PhD candidates work on the following competence areas - Research skills - Critical thinking: - Writing and communication - Ethical conduct - Leadership and collaboration - Teaching and mentoring	Yes	NA	Through the PhD writing process
The Netherlands	Yes	The PhD competence model which focuses on academic skills, personal development, and career orientation.	PhD candidates work on the following competence areas: (a) communication; (b) responsible conduct of science; (c) leadership and management; (d) research skills and knowledge; (e) teaching (f) professional development; (g) personal effectiveness.	Yes	The PhD competence model includes an assessment tool to identify competencies needed to develop and provides the possibility of monitoring PhD candidates' development.	Competence assessment is required at least annually (generally every 6 months; some require every 12 months) but recommended more frequently.

## Discussion

This study explores the current landscape of PhD nursing education in Finland, Lithuania, the Netherlands, and Kazakhstan, focusing on the organizational structure, entry criteria, supervision, degree requirements, and core competencies in nursing research and healthcare development. The findings reveal both shared features and context-specific differences among these national programs.

In line with the Bologna Process (European Commission, 2024), PhD nursing programs in the European countries studied are university-based and led by NSD chairs, typically professors with substantial expertise in nursing science and research. In contrast, Kazakhstan's PhD nursing programs are incorporated within broader faculties, like public health, reflecting the nascent development of nursing science in the country. Comparatively, several countries in East and Southeast Asia (ESEA) have longer-standing traditions of nursing science, which contributes to more mature doctoral programs (Molassiotis et al., 2020).

All countries require a master's degree for entry into PhD studies. The Netherlands additionally mandates strong English proficiency. Program duration ranges from 3 to 4 years, with Finland allowing extensions up to 8 years. These timeframes align with global patterns, where full-time PhD students typically take 3–5 years, and part-time students may take longer (Dobrowolska et al., 2021; Fang et al., 2016; Molassiotis et al., 2020; Nehls et al., 2016).

Funding structures also vary. In the Netherlands, PhD students are often salaried university employees, whereas in Lithuania and Finland, scholarships are selectively awarded. Kazakhstan offers state-funded positions. Limited funding is a known barrier, frequently compelling students to study part-time and delaying graduation (McKenna & Thompson, 2025). In ESEA, nearly half of institutions offer full financial support, with three-quarters covering research and conference expenses (Molassiotis et al., 2020).

Across all countries, doctoral nursing students must complete original research and coursework, typically including methodology. Teaching responsibilities are also common in the Netherlands and Kazakhstan. In ESEA, 85% of institutions require coursework in research methodology, theory development, and health promotion (Molassiotis et al., 2020). Given that many PhD-prepared nurses pursue academic careers, programs should offer courses enhancing faculty preparation and clinical competencies (Dobrowolska et al., 2021).

Supervision models differ significantly. European countries ensure that PhD students are guided by nursing science professors. On the contrary, Kazakhstan permits supervision by faculty without nursing science expertise. This gap stems from the limited number of PhD-prepared nurses available to supervise PhD nursing students, which may hinder the development of the discipline, echoing concerns raised in prior research (Kim et al., 2015). Supervisors without a nursing background often feel ill-equipped to mentor nursing students, particularly in qualitative research (Kayama et al., 2013). Given the central role of nursing scholars in advancing evidence-based practice, ensuring supervisors possess both content and methodological expertise is critical (Broome et al., 2023; Kim et al., 2022).

While all countries employ supervisory committees, the specificity of their roles varies. The Netherlands has well-defined standards, such as evaluating manuscripts based on originality and rigor, whereas Kazakhstan provides limited guidance. Notably, Kazakhstan mandates the inclusion of at least one international supervisor, a practice not consistently required elsewhere. Still, Finland involves international experts in thesis evaluation, reinforcing the value of global perspectives in enhancing research quality.

Requirements for graduation also vary. Finnish students must produce a thesis summary and three to four published papers or a monograph. Lithuanian students must author a monograph, publish two indexed articles, and present at two international conferences. In the Netherlands, the dissertation includes a general introduction, a range of peer-reviewed research articles forming a coherent line of inquiry, and a general discussion. Kazakhstan's requirement focuses on a defended dissertation with two to three publications. In ESEA, 75% of institutions demand publications, with 60% requiring international peer-reviewed journal articles (Molassiotis et al., 2020).

Developing a competent research workforce necessitates robust academic training. While all countries promote research, clinical, and teaching skills, only the Netherlands formally integrates these into a structured framework, the PhD Competence Model, covering academic and personal development, career planning, and self-assessment (Hammerslag et al., 2025; Stouthard & Cohen, 2016). Other countries lack formal evaluation tools for doctoral competencies. Numminen et al. (2019) defined essential competencies for PhD-prepared nurses, including research, ethics, leadership, communication, and intercultural competence, which have been validated as a self-assessment tool but require further study (Sterkenburg et al., 2025). Routine supervisor feedback and progress evaluations are essential for monitoring competency development and broader faculty growth (Numminen et al., 2019; Smaldone & Larson, 2021).

Faculty quality remains a cornerstone of PhD program success. In a study across seven countries, faculty quality was ranked as the most important determinant of doctoral education quality, ahead of program structure and resources (Kim et al., 2015). Mentorship and a supportive academic culture are fundamental to producing competent nursing educators and researchers (Kapucu & Bulut, 2019; Rollins Gantz & Hafsteinsdóttir, 2023).

Since obtaining independence in 1991, Kazakhstan adopted a clear prioritization process: first economic development, then political modernization. Kazakhstan experienced problems with the implementation of Bologna, including a lack of development of competency and performance standards, insufficient student choice and voice, and failure to meet the needs of employers. While there is evidence of gradual economic growth, however, in terms of political and public management reforms, still much needs to be done (Monobayeva & Howard, 2015).

The development of PhD nursing education in Kazakhstan is a multifaceted process influenced by cultural values, political will, and academic initiatives. Despite its autonomy, Kazakhstan continues to face infrastructure challenges in nursing research. Barriers include insufficient access to databases and journals, inadequate library services, and limited participation in international networks (Järvinen et al., 2023). Moreover, Kazakhstan's higher education environment is shaped by enduring cultural and political factors that directly influence the feasibility of PhD nursing education reforms. Historically, nursing has been regarded as an assistive role to medicine, a perception rooted in Soviet-era professional hierarchies. This cultural framing not only affects the public and policy valuation of nursing research but can also limit institutional willingness to allocate resources or grant autonomy to nursing faculties. Politically, the post-independence prioritization of economic growth over political modernization has meant that educational reforms often follow a top-down model with limited stakeholder consultation and questionable success at the program level (Lodhi & Ilyassova-Schoenfeld, 2023). As Monobayeva and Howard (2015) note, entrenched administrative cultures, centralized decision-making, and limited professional capacity within universities can stall or dilute implementation, even when policies are formally adopted. In the case of PhD nursing education, these features may lead to resistance from medical and other faculties, delays in establishing independent nursing departments, and a continued reliance on nonnursing supervisors. Similar patterns have been documented in other post-Soviet and Central Asian higher education reforms, where ambitious program designs faltered due to misalignment between policy objectives and institutional readiness. Without parallel investment in cultural change, political commitment, and implementation capacity, the recommendations outlined in this study risk partial or slow adoption. In the Central Asia region, additional gaps were identified in faculty qualifications, research alignment with national priorities, and accreditation standards (Kanzaki Izawa et al., 2021; Lechthaler et al., 2020; Llop-Gironés et al., 2024; WHO Regional Office for Europe, 2022).

Recruitment of PhD nursing candidates remains another concern for many universities. Factors such as misconceptions, lack of funding, and the perceived need for clinical experience deter applicants (Granner & Ayoola, 2021). Early engagement through research exposure and mentorship during undergraduate studies is essential (Stanfill et al., 2019). Structured support through training, mentorship, and conferences can facilitate smooth transitions into academic and clinical roles (Bice et al., 2019; Dobrowolska et al., 2021; Hafsteinsdóttir et al., 2020; Hølge-Hazelton et al., 2016; Stanfill et al., 2019). Leadership and mentoring programs for PhD students and PhD-prepared nurses (Hafsteinsdóttir et al., 2020) have demonstrated success in improving research, leadership, and decision-making skills among PhD nursing students and PhD-prepared nurses (van Dongen et al., 2021, 2023, 2024). These models illustrate how structured mentoring can build capacity for nursing leadership.

To strengthen PhD nursing education, governments and academic institutions must collaborate to develop well-funded, accessible programs that emphasize mentorship, practical research, and career development. Engaging stakeholders from universities to healthcare institutions can foster a pipeline of skilled nurse scientists, enhance healthcare delivery, and contribute meaningfully to public health. Co-ordinated action is required from universities, governments, and professional bodies to improve funding, supervision, inclusivity, and public perception of the nursing PhD. Strategies include early recruitment, better program design, and structural reforms such as recognizing PhD students as employees (McKenna & Thompson, 2025).

### Strengths and Limitations

Although this is the first study investigating and comparing PhD nursing education in the participating countries, the findings of the study should be carefully interpreted due to several limitations. Only four countries participated in the study: three West-European countries, Finland, Lithuania, and the Netherlands, and the Central-Asian Republic of Kazakhstan. While this may be seen as a limitation, the aim was to compare PhD education in these countries. Another limitation may be the fact that only one university per European country and two from Kazakhstan participated in the study, and data were collected through one contact person per university per country. The Kazakhstani two universities were the only two that had initiated a PhD program at the time of the data collection. On the other hand, the departments participating are small and therefore it can be expected that the contact persons were well informed about the situation in their respective university and country. The questionnaire used was self-developed, and only face validity was conducted, with no further psychometric testing being conducted. The questionnaire, however, was developed in line with the aims of the study, and no validated questionnaire was identified in the literature.

Although the data were collected in the year 2021, the findings are still considered to be relevant for doctoral education in the participating universities today. Although the generalizability of the findings is limited, the findings can be helpful for other nursing faculties of universities within the participating countries or other countries where doctoral nursing education and nursing science are in their early stages.

### Implication for Practice

Worldwide, the academic preparation of future nurses is crucial to meeting the health care needs of individuals, families, communities, and societies. PhD nursing education is essential for advancing research, nursing education, evidence-based practice, and a sustainable nursing workforce. PhD-prepared nurses generate, translate, and disseminate knowledge in academia and health care, educating and mentoring clinical nurses and shaping the future of nursing education. To ensure high-quality care and improved health outcomes, governments and policymakers must support PhD programs, fostering innovation, healthcare improvement, and informed policy development.

## Conclusion

This study examined PhD nursing education in Finland, Lithuania, and the Netherlands compared with Kazakhstan, in terms of program structure, entry requirements, supervision, requirements leading to the PhD, and academic competencies. While all countries offer research-oriented PhD programs, the European countries emphasize nursing expertise and share similar program structures, including rigorous entry standards and strong supervisory requirements. In contrast, Kazakhstan lacks independent PhD nursing programs, and supervisory qualifications often do not require advanced nursing expertise. These gaps limit the development of nursing science in the country.

To meet the future healthcare needs of the people of Kazakhstan, the government and policymakers need to support and strengthen PhD nursing education and expand the number of PhD-prepared nurses. Universities should establish independent, research-focused programs led by highly qualified nursing scholars, and PhD students should be supervised by PhD-prepared nurses across different areas of nursing science. Progress requires government and university support through effective policies, structures, and resources. However, as highlighted by Monobayeva and Howard (2015), limited professional capacity and incompatible administrative systems may hinder implantation. Therefore, raising awareness among the government, policy makers, nurses, and healthcare professionals about the importance of PhD nursing education, the role of nursing research, and evidence-based practice is critical. Efforts like these will enable the development of a greater pool of PhD-prepared nurses with strong competencies in conducting research, advancing evidence-based practice, and preparing an academically strong, vibrant, and sustainable nursing workforce that contributes to the health and well-being of the Kazakhstani population.

## Supplemental Material

sj-docx-1-son-10.1177_23779608251395097 - Supplemental material for Current State of PhD Nursing Education in Four Countries: A Comparative Analysis Supplemental material, sj-docx-1-son-10.1177_23779608251395097 for Current State of PhD Nursing Education in Four Countries: A Comparative Analysis by Thóra B. Hafsteinsdóttir, Joseph Almazan, Johanna Heikkilä, Lisbeth Fagerström and Aurelija Blaževičienė in SAGE Open Nursing

sj-docx-2-son-10.1177_23779608251395097 - Supplemental material for Current State of PhD Nursing Education in Four Countries: A Comparative Analysis Supplemental material, sj-docx-2-son-10.1177_23779608251395097 for Current State of PhD Nursing Education in Four Countries: A Comparative Analysis by Thóra B. Hafsteinsdóttir, Joseph Almazan, Johanna Heikkilä, Lisbeth Fagerström and Aurelija Blaževičienė in SAGE Open Nursing
