# Supplementary material for: Current State of PhD Nursing Education in Four Countries: A Comparative Analysis
Source: SAGE Open Nurs. 2025 Dec 23;11:23779608251395097. doi: 10.1177/23779608251395097 (PMC12739097; doi:10.1177/23779608251395097)
Supplement: sj-docx-1-son-10.1177_23779608251395097 - Supplemental material for Current State of PhD Nursing Education in Four Countries: A Comparative Analysis [file sj-docx-1-son-10.1177_23779608251395097.docx]

| **SUPPLEMENTARY FILE 1.** |  |
| --- | --- |
| **Current state of PhD Nursing Education in four countries: A comparative analysis** |  |

**Identification of best practices in management of PhD studies**

**Nursing Science Faculties**

Dear Colleague,

We would like to collect some information about organizational structure of the Nursing Science Department/Nursing School within your university, including the organization of PhD education offered to nurses in the universities participating in AccelEd. Please fill in this questionnaire on behalf of your department and provide us with all the information required. Some questions will require you to send extra files/documents in addition to the questionnaire. In such cases please send such files at the email address provided (preferably in Word format). Please send us the filled in questionnaire and the attached documents no later than April 3rd, 2021.

**University: ………………………………………………………………………………………**

**Faculty/Department of Nursing Science: …………………………………………………………………….**

**Nursing Science Department chair (name and surname): ………………………………………….**

**Date:…/……/2021**

1. **Organizational structure of the Nursing Science Department of your university:**

1. Under what organizational structure is Nursing Science Department (NSD) / or Nursing School (NS) of your organization managed?

a) NSD/ NS is a separate School under our medical university Yes/No

b) NSD/ NS is part of or managed by (please describe):…………………………………………... ……………………………………………………………………………………………………...

2. Please provide an organogram of the organizational model describing the Nursing Science Department/ the Nursing School within your Medical University (by sending it in a separate document attached with this questionnaire).

| Please provide a short description here: |
| --- |

3. What are the Vision and Mission of your NSD/ NS?

| Please provide a short description here: |
| --- |

4. Please describe the management team of your NSD/ NS: how many members, their positions, and educational background?

| Please provide a short description here: |
| --- |

5. Does your NSD have an annual plan/ strategic plan which describes objectives for the coming years? (Note: NSD/ NS plan could be part of the bigger plan) Yes/No

If that is the case, can you please describe the main aspects of the strategic plan?

| Please provide a short description here: |
| --- |

6. Your NSD/ NS oversees (please select):

a) Bachelor in NURSING educational program:

Yes/No IF NO WHICH FACULTY / DEPARTMENT oversees it:

………………………………………………………….

b) Master’s NURSING educational program:

Yes/No IF NO WHICH FACULTY / DEPARTMENT oversees it:

………………………………………………………….

c) PhD Nursing Science programs:

Yes/No, IF NO WHICH FACULTY / DEPARTMENT oversees it:

………………………………………………………………………………

d) Postgraduate nursing education:

Yes/No IF NO WHICH FACULTY / DEPARTMENT

oversees it: ……………………………………………………………………………….

**Organisational structure of PhD studies within the University:**

1. What is the organizational structure of PhD studies (for nurses) within the University?

| Please provide a short description: |
| --- |

1. Is the organization university or university of applied sciences?

| Please provide a short description here: |
| --- |

1. How many universities in your country award the PhD degree?

| Please provide a short description here: |
| --- |

1. Who is the head of the PhD programme in your country, - who is the PhD programme chair?

| Please provide a short description here: |
| --- |

1. What type of positions do PhD candidates have in your country and how are these positions funded?

| Please provide a short description here: |
| --- |

1. How many years does it take to complete PhD studies in your country?

| Please provide a short description here: |
| --- |

1. What is the content of the PhD programmes offered to nurses in your country?

| Please provide a short description here: |
| --- |

**Supervision of PhD students:**

1. Are PhD studies offered to nurses supervised by supervisory committees (Y/N)?

| Please provide a short description here: |
| --- |

1. Who are the members of the supervisory committee and what is their qualification?

| Please provide a short description here: |
| --- |

1. What are the minimum qualifications of supervisors in your country?

| Please provide a short description here: |
| --- |

1. Can you describe the role of the supervisory committee?

| Please provide a short description here: |
| --- |

**Requirements leading to the PhD degree:**

1. What are the requirements leading to the PhD degree?

| Please provide a short description here: |
| --- |

1. What are the requirements for the PhD thesis to include?

| Please provide a short description here: |
| --- |

1. At the end of PhD studies how many papers need to be published?

| Please provide a short description here: |
| --- |

1. Are there other requirements like presentations to be conducted or courses to be followed or other?

| Please provide a short description here: |
| --- |

**PhD core competences and how are they assessed:**

1. Are there specific PhD core competencies required to be developed?

| Please provide a short description here: |
| --- |

1. Are the PhD competencies assessed?

| Please provide a short description here: |
| --- |

1. Is Competence assessment tool used?

| Please provide a short description here: |
| --- |

1. Is competence assessment conducted through thesis assessment?

| Please provide a short description here: |
| --- |

**PhD assessment procedures and criteria:**

1. What procedures are used to assess PhD (how is the assessment conducted and by whom?)

| Please provide a short description here:  a) Who is responsible (leads the overall process) for the assessment procedures? Please describe and state the positions.  b) Who is conducting the assessment? |
| --- |

27. Does your department use assessment criteria to assess PhD theses?

| Please provide a short description here:  ___Yes ___NO  if NO, please explain:……………………………………………………….. |
| --- |

29. Please share or describe the criteria used to assess PhD theses.

| Please provide a short description here: |
| --- |

Thank you for taking the time to fill in this survey! We appreciate your contribution.

Best regards,

The research team.
